# Supplementary material for: A cross-sectional survey assessing the preparedness of the long-term care sector to respond to the COVID-19 pandemic in Ontario, Canada
Source: BMC Geriatr. 2020 Oct 22;20:421. doi: 10.1186/s12877-020-01828-w (PMC7578587; doi:10.1186/s12877-020-01828-w)
Supplement: Supplementary file 1 — Additional file 1. Online long-term care clinician survey questionnaire assessing the preparedness and readiness of the long-term care sector to respond to the COVID-19 pandemic. [file 12877_2020_1828_MOESM1_ESM.docx]

**Title: A cross-sectional survey assessing the preparedness of the long-term care sector to respond to the COVID-19 pandemic in Ontario, Canada**

**Corresponding Author:**

Henry Yu-Hin Siu, MSc MD CCFP(COE)^1^

1475 Upper Ottawa Street

Hamilton, Ontario, Canada

L8W 3J6

Email: [siuh3@mcmaster.ca](about:blank)

**Co-authors:**

Lorand Kristof, MSc MD CCFP(COE)^1,2^

Email: lorandkristof@yahoo.com

Dawn Elston, MA^1^

Email: elstond@mcmaster.ca

Abe Hafid, MPH^1^

Email: hafids3@mcmaster.ca

Fred Mather, MD CCFP FCFP^2^

Email: jfredmather@gmail.com

^1^Department of Family Medicine, McMaster University, Hamilton, Canada

5^th^ Floor, 100 Main Street West

Hamilton, Ontario Canada

L8P 1H6

^2^Ontario Long-Term Care Clinicians, Oshawa, Canada

202-1143 Wentworth Street West

Oshawa, Ontario, Canada

L1J 8P7

**ABSTRACT**

**Background:** The COVID-19 pandemic is a significant public health emergency that impacts all sectors of healthcare. The negative health outcomes for the COVID-19 infection have been most severe in the frail elderly dwelling in Canadian long-term care (LTC) homes.
**Methods:** An online cross-sectional survey of Ontario LTC Clinicians working in LTC homes in Ontario Canada was conducted to provide the LTC clinician perspective on the preparedness and engagement of the LTC sector during the COVID-19 pandemic. The survey questionnaire was developed in collaboration with the Ontario Long-Term Care Clinicians organization (OLTCC) and was distributed between March 30, 2020 to May 25, 2020. All registered members of the OLTCC and Nurse-led LTC Outreach Teams were invited to participate. The primary outcomes were: 1) the descriptive report of the screening measures implemented, communication and information received, and the preparation of the respondent’s LTC home to a potential COVID-19 outbreak; and 2) the level of agreement, as reported using a five-point Likert scale), to COVID-19 preparedness statements for the respondent’s LTC home was also assessed.

**Results:** The overall response rate was 54% (160/294). LTC homes implemented a wide range of important interventions (e.g. instituting established respiratory isolation protocols, active screening of new LTC admissions, increasing education on infection control processes, encouraging sick staff to take time off, etc). Ample communications pertinent to the pandemic were received from provincial LTC organizations, the government and public health officials. However, the feasibility of implementing public health recommendations, as well as the engagement of the LTC sector in pandemic planning were identified as areas of concern. Medical director status was associated with an increased knowledge of local implementation of interventions to mitigate COVID-19, as well as endorsing increased access to reliable COVID-19 information and resources to manage a potential COVID-19 outbreak in their LTC home.

**Conclusions:** This study highlights the communication to and implementation of recommendations in the Ontario LTC sector, despite some concerns regarding feasibility. Importantly, LTC clinician respondents clearly indicated that better engagement with LTC leaders is needed to plan a coordinated pandemic response.

**Key Words:** Long-term Care; COVID-19; Public Health; Pandemic planning

**BACKGROUND**

The COVID-19 pandemic is a significant public health emergency that impacts all sectors of healthcare. The negative health outcomes for the COVID-19 infection are most prevalent in the elderly. Current World Health Organization data report over 6 million cases and 370,000 deaths as of June 1, 2020.(1) Data from countries hardest hit by COVID-19 consistently report that the highest case fatality rate (CFR) are in adults over the age of 80 (13-20%).(2) This is in contrast the global case fatality rate of 6-7%.(2)

Older adults living in long-term care (LTC) are amongst the frailest in our society. The LTC setting is at high risk for the rapid spread of infection. Significant care needs and cognitive impairment makes the task of contact precautions and social isolation extremely difficult.(3-5) Underlying multi-morbidity, medical complexity, cognitive changes, and long-standing systemic issues resulting from chronic under-funding (e.g. for staffing, for addressing structural challenges such as the lack of private rooms) increase the risk of morbidity, loss of functional independence and mortality during a disease outbreak, such as influenza.(3, 6-8) Epidemiologic studies of the outbreak in King County, Washington reports a CFR in LTC of 33.7%.(9) However, in Ontario, Ministry of Health and Long-term care (MOHLTC) statistics from June 1, 2020 have confirmed 1,652 deaths in LTC residents; this represents 72% of all COVID-19 related deaths in Ontario.(10) At the peak of the pandemic within LTC in mid-May 2020, there were over 260 LTC homes with an active outbreak.(10) As a result, advocates, professional and LTC organizations, and even the Canadian Military have brought issues such as the state of disrepair of many LTC homes, the lack of private LTC resident rooms, nursing and personal support-worker shortages and training, lack of appropriate access to personal protective equipment (PPE), and long-standing under-funding for LTC homes starkly back into focus during this pandemic.(11-13)

In Ontario, the ongoing and significant communication between public health officials with primary and acute care leaders has resulted in a rapid response to try to mitigate the spread of COVID-19. Implementing community based COVID-19 assessment centers, delivering health care in virtually, updating case definitions and regular public health recommendation dissemination are some results of this engagement. However, the rapid spread of COVID-19 and the overwhelming rate of mortality observed in Ontario LTC homes raises important questions about the preparedness and readiness of, and the sufficiency and timeliness of communication with LTC homes during the pandemic. Therefore, understanding the preparedness and the engagement with the LTC sector during the COVID-19 pandemic should be systematically investigated. The Ontario Ombudsman will be investigating the Ontario government’s oversight of LTC homes during this pandemic,(14) and the Ontario government has announced the launching of an independent commission to review the LTC sector.(15) By undertaking a systematic cross-sectional survey of Ontario LTC clinicians, we hope to provide the clinician perspective on the preparedness for the pandemic through this study.

**METHODS**

This project was granted ethics approval by the Hamilton Integrated Research Ethics Board (Project #10760).

**Study Design and Setting**

We conducted a cross-sectional survey of clinicians from the Ontario Long-term Care Clinicians (OLTCC) and Nurse-led Outreach Teams (NLOTs) from March 30, 2020 to May 25, 2020 in Ontario, Canada. The OLTCC is a not-for-profit organization with a mission to provide advocacy and education to physicians, nurse practitioners, pharmacists and other LTC healthcare providers. NLOTs are teams of specialized nurses who provide consultative nursing services to LTC residents and support LTC home capacity building by optimizing access to ambulatory hospital services (e.g. reduction of potentially avoidable emergency department transfers, support end-of-life care provision in LTC homes, on-site rapid assessment of residents demonstrating clinical change). At the time of this study, the survey was sent to 294 clinicians (240 members of the OLTCC and 54 Nurse Practitioners, Registered Nurses or Allied Health professionals affiliated with NLOTs).

**Recruitment**

An anonymous electronic questionnaire was sent to all OLTCC and NLOT clinicians using the email distribution list of each organization.

**Questionnaire**

The COVID-19 pandemic response in LTC questionnaire was co-developed with study investigators that included two OLTCC board members (LK, FM). The final survey was subsequently approved by the executive board of the OLTCC prior to distribution amongst their membership. The survey consists of four sections. The first section collects demographic information about participants. The second section asks participants about screening measures, communication, information, and preparation for future outbreaks in their LTC home. The third section asks participants to rate their agreement with statements about information, communication, and preparation for COVID-19 in LTC on a five-point Likert scale (strongly disagree to strongly agree). The fourth section consists of 6 open-ended questions asking participants to reflect on the current COVID-19 pandemic, and the public health and government responses to the pandemic in LTC.

**Questionnaire Administration**

A modified Dillman method was used to maximize response rates.(16, 17) A study invitation letter was mailed out one week prior to the study information letter, which included a link to a digital survey hosted on the RedCap platform ([https://www.project-redcap.org/](about:blank)). Two subsequent email reminders were sent out two and four weeks following the survey link. An opt-in approach was used; all clinicians with an active email address received a survey link and were instructed to only complete the survey once. Completion, and submission of questionnaires implied a respondent’s consent to participate in the study. Questionnaires completed by May 25, 2020 were included in the analysis.

**Data Collection and Analysis**

Data from completed questionnaires were electronically collated using the RedCap platform. Planned subgroup analyses were performed for the following: age, sex, rurality status, medical director status, attending physician status, number of LTC homes a respondent worked in, the number of LTC residents under a respondent’s care, and the timeframe of survey completion (March 30 to April 22; and April 22 to May 25). LTC home rurality status was derived from the having a Rural Index of Ontario (RIO) score greater than 40.(18) The timeframe for survey completion was determined by the date set by MOHLTC in their directive restricted LTC staff to a single location of employment. Directives for visitor restrictions and active staff screening were in place as of March 30, 2020. While universal masking appears to have been officially mandated by April 15, 2020 in public health documents, many LTC homes had already enacted universal masking policies before this date. As a result, the authors were unable to identify a definitive date before which respondents’ LTC homes were not masking universally.

Respondent characteristics and other categorical variables were described using counts (proportions). Pearson’s Chi-Squared tests and Fischer’s Exact tests (for categorical data with expected cell sizes <5) were utilized to compare outcomes between exposure variables. Statistical significance was assessed by a two-tailed p-value of <0.05. Quantitative analyses were completed using IBM SPSS Statistics for Windows, Version 25.0.

**RESULTS**

The survey was distributed to 294 LTC clinicians across Ontario. The response rate was 54% (160/294); our survey item completion rate was 100%, except for 3 missing responses regarding rurality status. No surveys were removed from the final analysis. The results from the first three sections of the questionnaire are presented in this paper.

Half of the respondents identified as female (50.6%, 81/160). Respondents were largely physicians (80%, 128/160), worked in an urban region (87.3%, 137/160), older than 51 years of age (62.0%, 99/160), and identified their primary role as a medical director (59.4%, 95/160). Half of the respondents worked in only one LTC home (51.3%, 82/160), and 25.6% (41/160) reported working in LTC for more than 30 years (Table 1).

**Table 1: Profile of long-term care clinician respondents from Ontario, Canada.**

| **Variable** | **Overall Cohort (n= 160)** |
| --- | --- |
| Age, n (%) |  |
| 20 - 30 | 4 (2.5%) |
| 31 - 40 | 32 (20.0%) |
| 41 - 50 | 25 (15.6%) |
| 51 - 60 | 39 (24.4%) |
| 61 - 70 | 42 (26.3%) |
| >70 | 18 (11.3%) |
| Sex, n (%) |  |
| Male | 79 (49.4%) |
| Female | 81 (50.6%) |
| Profession, n (%) |  |
| Physician | 128 (80.0%) |
| Nurse Practitioner/Registered Nurse, Extended Class | 20 (12.5%) |
| Registered Nurse | 7 (4.4%) |
| Pharmacist | 5 (3.1%) |
| Respondent’s primary role(s) at LTC home, n (%)* |  |
| Medical Director | 95 (59.4%) |
| Attending Physician | 104 (65.0%) |
| Frontline Healthcare Worker | 5 (3.1%) |
| Local Leadership (e.g. Site Administrator, Director of Care, etc.) | 7 (4.4%) |
| Other | 21 (13.1%) |
| Number of years worked in LTC sector, n (%) |  |
| 1 - 5 | 32 (20.0%) |
| 6 - 10 | 32 (20.0%) |
| 11 - 20 | 29 (18.1%) |
| 21 - 30 | 26 (16.3%) |
| >30 | 41 (25.6%) |
| Number of long-term care homes worked in, n (%) |  |
| 1 | 82 (51.3%) |
| 2 | 41 (25.6%) |
| 3 | 15 (9.4%) |
| > 3 | 14 (8.8%) |
| N/A – Consultant Role | 8 (5.0%) |
| Number of residents routinely provide care for, n (%) |  |
| 0 - 25 | 17 (10.6%) |
| 26 - 50 | 34 (21.3%) |
| 51 - 75 | 19 (11.9%) |
| 76 - 100 | 18 (11.3%) |
| 101 - 150 | 30 (18.8%) |
| >150 | 31 (19.4%) |
| N/A - Consultant Role | 11 (6.9%) |
| Rurality status of respondents’ LTC home, n (%)† |  |
| Urban | 137 (87.3%) |
| Rural | 20 (12.7%) |

**Respondents were allowed to identify multiple roles. For example, a physician could serve as both a medical director and attending physician*

*† N = 157 due to incomplete survey responses.*

**Local LTC home pandemic response and communication**

The top five outbreak preventions measures implemented in respondent LTC homes were: 1) instituting established isolation protocols for respiratory conditions (92.5%, 148/160); 2) active screening of new LTC admissions (90.0%, 144/160); 3) increasing staff education about infection control processes (83.1%, 133/160); 4) active coordination with regional public health officials (83.1%, 133/160); and 5) encouraging sick staff members to take time off work (83.1%, 133/160). Thirty-eight respondents provided additional unlisted measures that were undertaken by their LTC home. Half of these respondents (50%, 19/38) indicated that visitor restrictions were also implemented. Other measures reported were active screening of staff and essential visitors (39.5%, 15/38), implementing virtual health solutions for patient care delivery (13.2%, 5/38), cohorting of LTC residents (10.5%, 4/38), increasing staffing levels (5.3%, 2/38), and coordination with acute care (5.3%, 2/38).

Respondents reported receiving significant communication regarding COVID-19 pandemic preparation from multiple sources, such as their professional associations (92.5%, 148/160), MOHLTC (90.0%, 144/160), and local public health authorities (80.6%, 129/160). In contrast, the head office of LTC operators (e.g. private corporations that own and run a chain of LTC homes) provided the least amount of communication (25.6%, 41/160) and were not deemed as sources of additional guidance (27.5%, 44/160) by respondents. Respondents indicated they would preferentially seek advice and guidance from local public health authorities (92.5%, 148/160), MOHLTC (63.8%, 102/160), and their professional associations (53.1%, 85/160) (Table 2).

*Insert Table 2 here*

There were no statistical differences in responses relating to instituted measures and communication based on individual characteristics of the sample or response timeframe except for medical director status. Regardless of medical director status, respondents overwhelmingly indicated that their LTC homes instituted active screening for new LTC admissions (89.5% vs. 90.8%, p=0.788), and to a lesser extent, provided increased updates to staff and LTC stakeholders (72.6% vs. 63.1%, p=0.200) (Table 2). For all other prevention measures, medical directors indicated a much higher rate of measure implementation in their LTC homes. The top five statistically different prevention measures were: 1) increased availability of hand sanitizer (91.6% vs. 69.2%; p= 0.000); 2) increased coordination with public health officials (92.6% vs. 69.2%; p= 0.000); 3) increased passive screening of visitors and staff (85.3% vs. 66.2%; p= 0.004); 4) instituted established respiratory isolation procedures (97.9% vs. 84.6%; p= 0.004); and 5) encouraged sick staff to take time off work (88.4% vs. 75.4%; p= 0.019). There were no statistical differences between medical director status for communication-focused responses.

**Engagement and Preparedness of with the LTC sector to manage a potential COVID-19 outbreak**

Overall, only 35.7% (57/160) of respondents agreed that the LTC sector was sufficiently engaged in planning a coordinated primary care response to COVID-19 outbreaks in the community (Table 3). While 76.3% (122/160) of respondents agreed with local public health recommendations for LTC; only 54.4% (87/160) agreed that the recommendations were feasible for LTC. When asked if their LTC home is ready to manage a potential COVID-19 outbreak, 31.9% (51/160) of respondents were neutral or chose not to respond; combined with the 19.4% (31/160) of respondents that indicated disagreement, over half of respondents (51.3%, 82/160) were unsure or felt their LTC home could manage a COVID-19 outbreak. Sufficient resource availability on-site to manage an outbreak was a concern for 30% (48/160) of respondents. Furthermore, only 31.9% (51/160) of respondents agreed that securing additional resources during an outbreak would be possible.

*Insert Table 3 here*

There were no statistical differences in responses relating to LTC pandemic preparedness or engagement in planning based on individual characteristics of the sample or response timeframe except for medical director status. Specifically, medical directors indicated more agreement that their LTC homes had access to reliable sources of information about COVID-19 to educate staff and the public (p=0.027), and that their LTC homes had the resources on-site to manage a COVID-19 outbreak (Table 4).

*Insert Table 4 here*

**DISCUSSION**

We present in this study the results of a systematic survey of Ontario LTC clinicians about the communication, preparedness, and engagement of the LTC sector during the COVID-19 pandemic. Overall, our survey reports the widespread implementation of several important interventions in LTC homes during the COVID-19 pandemic (e.g. instituting established respiratory isolation protocols, active screening of new LTC admissions, actively coordinating with regional public health and encouraging sick staff members to take time of work). Respondents also felt that there was ample communication pertinent to the pandemic from provincial LTC organizations, the government and public health officials. However, the feasibility of implementing these public health recommendations in LTC was a concern.

Most importantly, our respondents indicated that the engagement of the LTC sector in a coordinated community-based primary care response was lacking. This indicates a strong recommendation that expert LTC clinicians need to be invited and engaged early in the process of any future planning for coordinated community response. Developing and strengthening the working relationship between the LTC sector and the government should be a key priority. This could result in the development of future preventive measures that are acceptable, feasible, rapidly spreadable, and informed by clinical evidence and experience. As it was so eloquently stated in an earlier publication, a successful response and management of COVID-19 in LTC relies not only on rapid diagnosis, the ability to manage the clinical manifestations in LTC, early initiation of policies to mitigate and prevent future spread; but requires the awareness of key members in the LTC sector about the decisions being made by various levels of government.(19)

Interestingly, respondents indicated that LTC operators provided the least amount of communication and were unlikely sources of additional guidance during the pandemic. LTC outbreaks in the Washington county, as well as the reports out of Europe were harbingers of what would be inevitable in Ontario. LTC operators could have taken this opportunity to provide strong leadership to their LTC homes during this time. Our study results highlight an important future opportunity for LTC operators to play a more essential role in protecting their residents and ensuring that their LTC homes could successfully and feasibly implement public health recommendations.

The authors have defined “preparedness” in this questionnaire as a function of three factors: 1) timeliness and appropriateness of recommendation communication (Tables 3 and 4, statements 1-4); 2) resources available to manage and respond to the changing demands of the pandemic (Tables 3 and 4, statements 5 - 7); and 3) perception of LTC sector engagement (Tables 3 and 4, statements 8). Not surprising, and consistent with the responses received in section 2 of the questionnaire, the lowest level of neutral responses was for statements 2 and 3, which inquired about the reliability and relevancy of the information received.

The statements with the highest proportion of neutral and “prefer not to respond/unsure” responses were for statements 6 and 7. These two statements inquired about the ability of the LTC home to respond to the growing resource demand in the event of a pandemic (statement 6), as well as a respondent’s confidence in their own LTC home to manage an outbreak (statement 7). In the context of these two statements, a high rate of neutral responses in combination compounds the rate of disagreeing responses; this indicates that respondents did not feel their LTC home had the ability to secure additional resources or manage a COVID-19 outbreak. This finding highlights the need to address the underlying issues (e.g. chronic under-funding, inadequate staffing, and the physical environment of LTC homes) that increase the vulnerability of the LTC sector to the pandemic. Mounting a successful future coordinated LTC outbreak management response will first require a meaningful collaboration between all LTC stakeholders (e.g. LTC residents and families, LTC clinicians, politicians, advocacy groups, private corporations, and others) to address the complex systemic challenges inherent to this sector.

The authors had hypothesized a priori that factors such as rurality, the number LTC homes and residents under a respondent’s care, the timeframe of response, and medical director status could impact responses. However, of all these variables, only medical director status was found to result in statistically different responses in the questionnaire. Medical directors have a unique role in the LTC home; they sit with LTC home leadership regularly to ensure high quality care is being provided to LTC residents. Medical directors would have numerous opportunities to be aware, reminded and up to date with the regular public health communications, and which mitigation strategies were implemented in their LTC homes. On the other hand, attending physicians may not be as up to date on the local policies and recommendations being implemented within the home during this time. We hypothesize that this knowledge gap could have been the result of inconsistent communication between the LTC leadership and attending physicians and frontline staff; this could explain the statistical difference observed in our survey responses. As a result, establishing a more efficient and effective communication structure within individual LTC homes could have more effectively disseminated vital knowledge to frontline clinicians not in positions of leadership during the COVID-19 pandemic.

**Strengths and Limitations**

This study has several strengths that support the validity of our results. First, our survey was co-developed with a key LTC stakeholder and organization in Ontario. This approach allowed the OLTCC to review and direct the information to be collected. Second, the response and item completion rate for a survey of this length is very robust, especially during a period where LTC clinicians have significant priorities to juggle. Third, respondent demographics represented a diverse LTC clinician population, which included an equal split amongst male and female providers, and good representation across the age and LTC work experience spectrum. Lastly, it should be noted that respondents used the entire range of the Likert scale in their responses; no ceiling or floor effect were noted in section 3 of the questionnaire.

The authors also acknowledge several limitations. First, although we have a good response rate for an online questionnaire study, our respondents were mainly physicians in urban areas. Frontline nursing staff and clinicians from rural settings are under-represented in our respondent population. Our sample was also older, as over half of the respondents were above the age of 51; however, previous Canadian data which reported an average age of 52.4 years.(20) Second, LTC clinicians that are actively affiliated with a professional LTC association would be more interested in attending continuing medical education events and review the regular updates being sent during the COVID-19 pandemic. As a result, these clinicians may be more aware of pandemic mitigation strategies, and more willing to complete an online questionnaire. Therefore, the results presented here may not represent the opinions of those LTC clinicians that are not members of professional associations. Third, while our data suggest that public health interventions were widely implemented, our questionnaire was not designed to demonstrate whether these interventions were meaningful enough to prevent a potential individual outbreak. Similarly, our survey does not assess the extent to which our respondents felt that public health interventions could result in potentially negative impacts on their LTC residents. It is important to note that recommended public health interventions could result in worsening cognition and functional decline because of the restriction of external visitors and in-home recreational activities.(21) This negative impact ultimately lowers the quality of life for LTC residents, and should to be considered when planning future pandemic responses in LTC.

Lastly, the survey was created and distributed during a rapidly changing LTC landscape during the COVID-19 pandemic. Because recommendations from public health and local government officials were being communicated daily, responses from later respondents may be different than earlier respondents. To mitigate this limitation, we identified key recommendations that could impact the nature of responses (e.g. LTC staff have one work location, visitor restrictions, active staff screening, masking protocols). Visitor restrictions and active screening recommendations pre-dated the survey distribution. Masking protocols were implemented variably and did not correspond necessarily to the date of the mandatory universal masking directive. As a result, the authors decided to use the recommendation date (i.e. LTC staff being restricted to a single location of work - April 22, 2020), because this resulted in two distinct respondent cohorts, with sufficient number of responses, to allow for a formal sub-analysis.

**CONCLUSION**

In Ontario, the LTC setting and LTC residents were hardest hit by COVID-19. The disproportionate death toll in LTC highlights the need for a better understanding of the real-time experiences of LTC clinicians during the first wave of this pandemic. The results of this study do highlight the work and effort made by public health and governmental authorities to communicate information to the LTC sector. However, unilateral communication is limited especially during a pandemic, and LTC clinician respondents clearly indicated that better engagement with LTC leaders during the planning of a coordinated pandemic response is needed. Further study is needed to understand the reasons behind this perception, what could be done to improve engagement, and would be vital to allow the healthcare sector respond better and earlier to subsequent rounds of COVID-19 infection in the community and ultimately in LTC.

**LIST OF ABBREVIATIONS**

CFR - Case Fatality Rate

LTC - Long-term Care

MOHLTC - Ministry of Health and Long-term Care

PPE - personal protective equipment

OLTCC - Ontario Long-term Care Clinicians

NLOT - Nurse-led Outreach Team

**DECLARATIONS**

*Ethics approval and consent to participate*

This project was granted ethics approval by the Hamilton Integrated Research Ethics Board (Project #10760). Voluntary completion and submission of responses confirmed implied consent to participate in this survey study

*Consent for Publication*

Not Applicable

*Availability of data and materials*

The datasets used and/or analysed during the current study are available from the corresponding author on reasonable request.

*Competing Interests*

Authors HS, DE, AH have no competing interests to declare. Author LK is a current board director of the Ontario Long-Term Care Clinicians organization. Author FM is the current president of the Ontario Long-Term Care Clinicians organization.

*Funding*

Not Applicable

*Authors’ contributions*

Authors HS, LK, DE, AH, FM were substantively involved with the conception of the work, as well as data interpretation, and drafting the final version to be published. All authors have read and approved the final version of the manuscript. In addition to this, authors HS, DE, AH were substantively involved with the data acquisition and analysis. All authors agree to accountable to the aspects of the work relating to the accuracy or integrity of the work reported in this paper.

*Acknowledgements*

Not applicable

**REFERENCES**

1. WHO. Coronavirus disease (COVID-19) Situation Report. 2020. Report No.: 133.

2. Roser M, Ritchie H, Ortiz-Ospina E, Hasell J. Mortality Risk of COVID-19 2020 [Available from: <https://ourworldindata.org/mortality-risk-covid#case-fatality-rate-of-covid-19-by-age>.

3. Dosa D, Jump RLP, LaPlante K, Gravenstein S. Long-Term Care Facilities and the Coronavirus Epidemic: Practical Guidelines for a Population at Highest Risk. Journal of the American Medical Directors Association. 2020.

4. Strausbaugh LJ, Sukumar SR, Joseph CL. Infectious disease outbreaks in nursing homes: an unappreciated hazard for frail elderly persons. Clin Infect Dis. 2003;36(7):870-6.

5. Gardner W, States D, Bagley N. The Coronavirus and the Risks to the Elderly in Long-Term Care. J Aging Soc Policy. 2020:1-6.

6. Barker WH, Borisute H, Cox C. A study of the impact of influenza on the functional status of frail older people. Archives of internal medicine. 1998;158(6):645-50.

7. Wang H, Li T, Barbarino P, Gauthier S, Brodaty H, Molinuevo JL, et al. Dementia care during COVID-19. Lancet. 2020;395(10231):1190-1.

8. RNAO. Long-Term Care Systemic Failings: Two Decades of Staffing and Funding Recommendations. 2020 June 5, 2020.

9. McMichael TM, Clark S, Pogosjans S, Kay M, Lewis J, Baer A, et al. COVID-19 in a Long-Term Care Facility - King County, Washington, February 27-March 9, 2020. MMWR Morb Mortal Wkly Rep. 2020;69(12):339-42.

10. MOHLTC O. <https://www.ontario.ca/page/how-ontario-is-responding-covid-19#section-0> 2020 [

11. Holroyd-Leduc JM, Laupacis A. Continuing care and COVID-19: a Canadian tragedy that must not be allowed to happen again. CMAJ. 2020.

12. OLTCC. COVID-19 News 2020 [Available from: <https://www.oltcc.ca/news>.

13. CAF. JTFC Observations in Long Term Care Facilities in Ontario. 2020 May 20, 2020.

14. Ontario Ombudsman to investigate government's oversight of Long-Term Care homes during pandemic [press release]. Ombudsman Ontario, June 1, 2020 2020.

15. Ontario announces independent commission into Long-Term Care [press release]. May 19, 2020 2020.

16. Dillman D. Mail and Internet Surveys: The Tailored Design Method. New York, NY: Wiley; 2000.

17. Schaefer DR, Dillman D. Development of a Standard E-Mail Methodology: Results of an Experiment. Public Opin Q. 1998;62(3).

18. Glazier RH, Zagorski BM, BRayner J. Comparison of Primary Care Models in Ontario by Demographics, Case Mix and Emergency Department Use, 2008/09 to 2009/10.; 2012.

19. D'Adamo H, Yoshikawa T, Ouslander JG. Coronavirus Disease 2019 in Geriatrics and Long-Term Care: The ABCDs of COVID-19. Journal of the American Geriatrics Society. 2020;68(5):912-7.

20. Lam JM, Anderson GM, Austin PC, Bronskill SE. Family physicians providing regular care to residents in Ontario long-term care homes: characteristics and practice patterns. Can Fam Physician. 2012;58(11):1241-8.

21. Mok VCT, Pendlebury S, Wong A, Alladi S, Au L, Bath PM, et al. Tackling challenges in care of Alzheimer's disease and other dementias amid the COVID-19 pandemic, now and in the future. Alzheimers Dement. 2020.

**Table 2: Respondents' experiences and views on COVID19 preparation, per Medical Director status (n= 160)**

| V**ariable** | **Overall Cohort**  **(n= 160)** | **Primary Role: Medical Director** | | |
| --- | --- | --- | --- | --- |
|  |  | **Yes**  **(n= 95)** | **No**  **(n= 65)** | **P-Value*** |
| **Measures currently instituted in respondents’ LTC home during the COVID19 pandemic, n (%):** |  |  |  |  |
| Passive screening of visitors and staff | 124 (77.5%) | 81 (85.3%) | 43 (66.2%) | 0.004 |
| Active screening of new admissions | 144 (90.0%) | 85 (89.5%) | 59 (90.8%) | 0.788 |
| Increased availability of personal protective equipment (PPE) | 127 (79.4%) | 81 (85.3%) | 46 (70.8%) | 0.026 |
| Increased availability of hand sanitizer | 132 (82.5%) | 87 (91.6%) | 45 (69.2%) | 0.000 |
| Encouraging sick staff to take time off work | 133 (83.1%) | 84 (88.4%) | 49 (75.4%) | 0.019 |
| Increased staff education about infection control processes | 133 (83.1%) | 84 (88.4%) | 49 (75.4%) | 0.031 |
| Instituted established isolation protocols for respiratory conditions | 148 (92.5%) | 93 (97.9%) | 55 (84.6%) | 0.004^a^ |
| Increased updates to staff and long-term care stakeholders | 110 (68.8%) | 69 (72.6%) | 41 (63.1%) | 0.200 |
| Active coordination with public health officials | 133 (83.1%) | 88 (92.6%) | 45 (69.2%) | 0.000 |
| Other | 38  (23.8%) | 20 (21.1%) | 18 (27.7%) | 0.332 |
| **Sources from which respondents received knowledge and communication around procedures for preparing COVID19, n (%):** |  |  |  |  |
| Ontario Ministry of Health and Long-Term Care | 144 (90.0%) | 83 (87.4%) | 61 (93.8%) | 0.180 |
| Health Canada | 73 (45.6%) | 39 (41.1%) | 34 (52.3%) | 0.160 |
| Local Public Health authorities | 129 (80.6%) | 79 (83.2%) | 50 (76.9%) | 0.327 |
| Professional associations | 148 (92.5%) | 91 (95.8%) | 57 (87.7%) | 0.070^a^ |
| Long-term care corporate head office | 41 (25.6%) | 29 (30.5%) | 12 (18.5%) | 0.086 |
| Other long-term care homes | 34 (21.3%) | 25 (26.3%) | 9 (13.8%) | 0.058 |
| Other | 45 (28.1%) | 30 (31.6%) | 15 (23.1%) | 0.240 |
| **From whom would respondents seek guidance if a COVID-19 outbreak occurred in their LTC home, n (%):** |  |  |  |  |
| Ontario Ministry of Health and Long-Term Care | 102 (63.8%) | 59 (62.1%) | 43 (66.2%) | 0.601 |
| Health Canada | 39 (24.4%) | 18 (18.9%) | 21 (32.3%) | 0.053 |
| Local Public Health authorities | 148 (92.5%) | 91 (95.8%) | 57 (87.7%) | 0.070 **^a^** |
| Professional associations | 85 (53.1%) | 52 (54.7%) | 33 (50.8%) | 0.621 |
| Long-term care corporate head office | 44 (27.5%) | 26 (27.4%) | 18 (27.7%) | 0.964 |
| Other long-term care homes | 33 (20.6%) | 23 (24.2%) | 10 (15.4%) | 0.175 |
| Other | 35 (21.9%) | 22 (23.2%) | 13 (20.0%) | 0.635 |
| **Pearson's Chi-Square Tests*  *^a^Fischer's Exact test.* |  |  |  |  |

**Table 3: Respondents' level of agreement to COVID19 preparedness statements (n= 160)**

| **Preparedness Statement** | **Strongly Agree** | **Agree** | **Neither Agree nor Disagree** | **Disagree** | **Strongly Disagree** | **Do Not Know/Prefer Not to Answer** |
| --- | --- | --- | --- | --- | --- | --- |
|  | **N (%)** | **N (%)** | **N (%)** | **N (%)** | **N (%)** | **N (%)** |
| 1. My LTC home has received sufficient and timely communication and information from government and public health sources to prepare my LTC for managing a potential COVID19 outbreak. | 34 (21.3%) | 61 (38.1%) | 23 (14.4%) | 23 (14.4%) | 11 (6.9%) | 8 (5.0%) |
| 1. My LTC home had access to reliable sources of information about COVID19 to educate staff and the public. | 38 (23.8%) | 81 (50.6%) | 15 (9.4%) | 12 (7.5%) | 6 (3.8%) | 8 (5.0%) |
| 1. Recommendations made by the local public health authority for front-line healthcare workers are relevant for LTC. | 52 (32.5%) | 70 (43.8%) | 12 (7.5%) | 19 (11.9%) | 6 (3.8%) | 1 (0.6%) |
| 1. Recommendations made by the local public health authority for front-line healthcare workers are feasible for LTC. | 22 (13.8%) | 65 (40.6%) | 26 (16.3%) | 36 (22.5%) | 7 (4.4%) | 4 (2.5%) |
| 1. My LTC home has the resources on-site required to manage a COVID19 outbreak. | 13 (8.1%) | 56 (35.0%) | 35 (21.9%) | 37 (23.1%) | 11 (6.9%) | 8 (5.0%) |
| 1. My LTC home would be able to secure additional resources required in the event of a COVID19 outbreak. | 15 (9.4%) | 36 (22.5%) | 44 (27.5%) | 24 (15.0%) | 10 (6.3%) | 31 (19.4%) |
| 1. My LTC home is ready to manage a COVID19 outbreak. | 13 (8.1%) | 65 (40.6%) | 38 (23.8%) | 23 (14.4%) | 8 (5.0%) | 13 (8.1%) |
| 1. The LTC sector was sufficiently engaged in planning a coordinated primary care response to a COVID19 outbreak in the community. | 14 (8.8%) | 43 (26.9%) | 38 (23.8%) | 35 (21.9%) | 20 (12.5%) | 10 (6.3%) |

**Table 4: Respondents' level of agreement to COVID19 preparedness statements based on Medical Director status (n= 160)**

| **Preparedness Statement** | **Medical Director (Y/N)** | **Strongly Agree** | **Agree** | **Neither Agree nor Disagree** | **Disagree** | **Strongly Disagree** | **Do Not Know/Prefer Not to Answer** | **P-Value*** |
| --- | --- | --- | --- | --- | --- | --- | --- | --- |
|  |  | **N (%)** | **N (%)** | **N (%)** | **N (%)** | **N (%)** | **N (%)** |  |
| 1. My LTC home has received sufficient and timely communication and information from government and public health sources to prepare my LTC for managing a potential COVID19 outbreak. | Yes (n= 95) | 23 (24.2%) | 37 (38.9%) | 13 (13.7%) | 13 (13.7%) | 7 (7.4%) | 2 (2.1%) | 0.417 |
|  | No (n= 65) | 11 (16.9%) | 24 (36.9%) | 10 (15.4%) | 10 (15.4%) | 4 (6.2%) | 6 (9.2%) |  |
| 1. My LTC home had access to reliable sources of information about COVID19 to educate staff and the public. | Yes (n= 95) | 25 (26.3%) | 48 (50.5%) | 9 (9.5%) | 6 (6.3%) | 6 (6.3%) | 1 (1.1%) | 0.027 |
|  | No (n= 65) | 13 (20.0%) | 33 (50.8%) | 6 (9.2%) | 6 (9.2%) | 0 (0.0%) | 7 (10.8%) |  |
| 1. Recommendations made by the local public health authority for front-line healthcare workers are relevant for LTC. | Yes (n= 95) | 33 (34.7%) | 41 (43.2%) | 5 (5.3%) | 13 (13.7%) | 3 (3.2%) | 0 (0.0%) | 0.518 |
|  | No (n= 65) | 19 (29.2%) | 29 (44.6%) | 7 (10.8%) | 6 (9.2%) | 3 (4.6%) | 1 (1.5%) |  |
| 1. Recommendations made by the local public health authority for front-line healthcare workers are feasible for LTC. | Yes (n= 95) | 14 (14.7%) | 41 (43.2%) | 14 (14.7%) | 21 (22.1%) | 4 (4.2%) | 1 (1.1%) | 0.744 |
|  | No (n= 65) | 8 (12.3%) | 24 (36.9%) | 12 (18.5%) | 15 (23.1%) | 3 (4.6%) | 3 (4.6%) |  |
| 1. My LTC home has the resources on-site required to manage a COVID19 outbreak. | Yes (n= 95) | 12 (12.6%) | 34 (35.8%) | 23 (24.2%) | 16 (16.8%) | 8 (8.4%) | 2 (2.1%) | 0.008 |
|  | No (n= 65) | 1 (1.5%) | 22 (33.8%) | 12 (18.5%) | 21 (32.3%) | 3 (4.6%) | 6 (9.2%) |  |
| 1. My LTC home would be able to secure additional resources required in the event of a COVID19 outbreak. | Yes (n= 95) | 13 (13.7%) | 19 (20.0%) | 29 (30.5%) | 12 (12.6%) | 6 (6.3%) | 16 (16.8%) | 0.158 |
|  | No (n= 65) | 2 (3.1%) | 17 (26.2%) | 15 (23.1%) | 12 (18.5%) | 4 (6.2%) | 15 (23.1%) |  |
| 1. My LTC home is ready to manage a COVID19 outbreak. | Yes (n= 95) | 11 (11.6%) | 40 (42.1%) | 22 (23.2%) | 14 (14.7%) | 5 (5.3%) | 3 (3.2%) | 0.058 |
|  | No (n= 65) | 2 (3.1%) | 25 (38.5%) | 16 (24.6%) | 9 (13.8%) | 3 (4.6%) | 10 (15.4%) |  |
| 1. The LTC sector was sufficiently engaged in planning a coordinated primary care response to a COVID19 outbreak in the community. | Yes (n= 95) | 10 (10.5%) | 29 (30.5%) | 20 (21.1%) | 18 (18.9%) | 14 (14.7%) | 4 (4.2%) | 0.305 |
|  | No (n= 65) | 4 (6.2%) | 14 (21.5%) | 18 (27.7%) | 17 (26.2%) | 6 (9.2%) | 6 (9.2%) |  |
| **** Fischer's Exact test****.* | | | | | | | | |
